# Innovations in Workforce Education for Family Caregiving

**DOI:** 10.1093/geroni/igab046.1552

**Published:** 2021-12-17

**Authors:** Kathryn Sexson, Jennifer Mongoven, Lisa Badovinac, Theresa Harvath

**Affiliations:** 1 University of California, Davis, UC Davis, California, United States; 2 University of California, Davis, Sacramento, California, United States

## Abstract

The number of adults providing care to a family member in the US is estimated at more than 50 million, with nearly half of those individuals providing complex care. National organizations, such as AARP, and federal programs, such as the Geriatric Workforce Enhancement Program, have identified the family caregiver as an integral member of the health care team, yet there is a paucity of clinical workforce education programs for how best to partner and support family caregivers. A virtual summit was held in September 2020 to highlight existing educational programs designed to prepare undergraduate and graduate health professional students or practicing clinicians in their efforts to support family caregivers. The meeting consisted of a keynote, 6 podium presentations and 12 poster presentations. Primary themes emerged around target learners, curricula topics, and outcomes. Programs targeted learners from across the workforce, from undergraduate students to continuing professional education programs, with the majority targeting graduate (masters and doctoral) learners. Several programs were interprofessional in development, delivery and target learner. Curricula topics varied across programs and included caregiver assessments, multicultural considerations, communication, care plan development and risk screening. Education outcomes primarily focused on assessment of participants’ confidence and knowledge. The summit highlighted that the topic of family caregiving is included in clinical education inconsistently, if at all. The summit helped identify gaps in education, curriculum development, and the need for common learning outcomes to strengthen a clinician’s ability to support family caregivers as part of the care team.

